# Investigation of New Morpholino Oligomers to Increase Survival Motor Neuron Protein Levels in Spinal Muscular Atrophy

**DOI:** 10.3390/ijms19010167

**Published:** 2018-01-06

**Authors:** Agnese Ramirez, Sebastiano G. Crisafulli, Mafalda Rizzuti, Nereo Bresolin, Giacomo P. Comi, Stefania Corti, Monica Nizzardo

**Affiliations:** Dino Ferrari Centre, Neuroscience Section, Department of Pathophysiology and Transplantation (DEPT), University of Milan, Neurology Unit, IRCCS Foundation Ca’ Granda Ospedale Maggiore Policlinico, via Francesco Sforza 35, 20122 Milan, Italy; agnese.ramirez@gmail.com (A.R.); sebastiano.crisafulli@gmail.com (S.G.C.); mafalda.rizzuti@gmail.com (M.R.); nereo.bresolin@unimi.it (N.B.); giacomo.comi@unimi.it (G.P.C.); stefania.corti@unimi.it (S.C.)

**Keywords:** spinal muscular atrophy, morpholino, therapy

## Abstract

Spinal muscular atrophy (SMA) is an autosomal-recessive childhood motor neuron disease and the main genetic cause of infant mortality. SMA is caused by deletions or mutations in the survival motor neuron 1 (*SMN1*) gene, which results in SMN protein deficiency. Only one approved drug has recently become available and allows for the correction of aberrant splicing of the paralogous *SMN2* gene by antisense oligonucleotides (ASOs), leading to production of full-length SMN protein. We have already demonstrated that a sequence of an ASO variant, Morpholino (MO), is particularly suitable because of its safety and efficacy profile and is both able to increase SMN levels and rescue the murine SMA phenotype. Here, we optimized this strategy by testing the efficacy of four new MO sequences targeting *SMN2*. Two out of the four new MO sequences showed better efficacy in terms of SMN protein production both in SMA induced pluripotent stem cells (iPSCs) and SMAΔ7 mice. Further, the effect was enhanced when different MO sequences were administered in combination. Our data provide an important insight for MO-based treatment for SMA. Optimization of the target sequence and validation of a treatment based on a combination of different MO sequences could support further pre-clinical studies and the progression toward future clinical trials.

## 1. Introduction

Spinal muscular atrophy (SMA) is an autosomal-recessive neurodegenerative disease with an infantile or early onset and represents the main genetic cause of infant mortality and the second most common autosomal-recessive disease in the Caucasian population. SMA is caused by a homozygous mutation of the survival motor neuron 1 (*SMN1*) gene, which results in reduced levels of the functional SMN protein [1]. SMA is characterized by progressive loss of motor neurons (MNs) in the ventral horns of the spinal cord, which causes progressive muscle weakness, paralysis and premature death [2]. 

In humans, two nearly identical copies of the *SMN* gene are located on chromosome 5q: telomeric *SMN1* and centromeric *SMN2*. The latter differs from *SMN1* by a single C > T nucleotide substitution within the coding region, precisely within exon 7 (c.840 C > T) [3], which causes alternative splicing of *SMN2* and the removal of exon 7 from 90% of *SMN2* mature mRNAs [4]. The SMN protein that lacks exon 7 (SMNΔ7) is not functional, has a reduced ability to oligomerize and is susceptible to rapid degradation [5]. Only 10% of *SMN2* mRNAs retain exon 7 and produce functional SMN protein. Therefore, the number of *SMN2* gene copies present in the genome is the predominant modulator of the SMA clinical phenotype and is inversely correlated with the severity of the disease. A majority of the current SMA therapeutic approaches are focused on increasing exon 7 retention in *SMN2*, including Nusinersen, which is the only compound that has been recently approved by the FDA/EMA (www.curesma.com). However, many unknown factors regarding Nusinersen treatment must be clarified, such as its efficacy in different types of patients and in symptomatic cases as well as the effects of repeated invasive injections. 

Exon 7 retention in *SMN2* mRNAs depends on the effects of different flanking splicing regulatory sites. Antisense oligonucleotides (ASOs) are an effective tool that can act on these splicing regulatory sequences, particularly on splicing silencers, thus enhancing exon 7 retention and the production of full-length SMN protein [6,7,8]. Two types of ASOs have been and are extensively used in preclinical and clinical studies. 2’-*O*-methyl-modified phosphorothioate (2OMePS) oligonucleotides and the more stable variant 2’-*O*-(2-methoxyethyl)-modified (MOE) phosphorothioate oligonucleotides belong to the first class, while the second class consists of morpholino oligomers (MOs), which have a good safety and efficacy profile.

Many groups have demonstrated that targeting the intronic splicing silencer N1 (ISS-N1) downstream of exon 7 with ASOs can efficiently promote exon 7 inclusion, which increases the production of full-length SMN protein and significantly extends the survival of SMA mice [9,10]. In particular, we demonstrated the efficacy of an ASO sequence of 25-nucleotides with an MO chemical structure that targets the ISS-N1 region within *SMN2* (HSMN2Ex7D (-10-34)) (MO-10-34) [11]. Following a single bolus injection at postnatal day 0, MO-10-34 efficiently increases the levels of functional SMN protein and leads to a robust improvement of neuromuscular pathologic hallmarks, improved motor performance and increased survival up to 30-fold compared to untreated affected littermates [11]. 

Here, we investigated whether the efficacy of MO treatment could be increased by testing new MO sequences that target different regions within *SMN2* ISS-N1 downstream of exon 7 when administered either alone or in combination in human wild-type cells, SMA cells and/or SMA mice. We demonstrated that two out of the four new sequences significantly increased SMN production in vitro and in vivo when administered alone and even more when administered in combination.

## 2. Results

### 2.1. Upregulation of SMN in Wild-Type Cells

**Figure 1:**
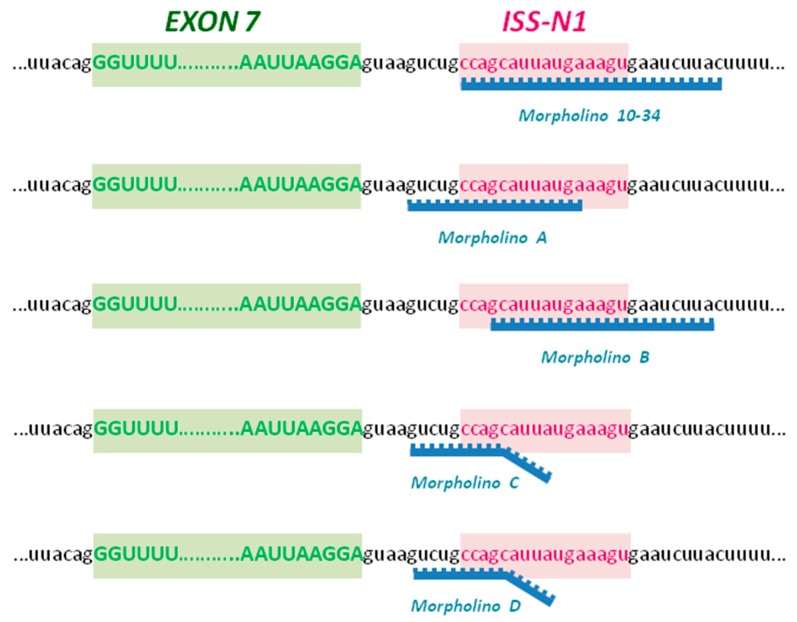
Morpholino (MO) sequences design. We designed four new MO sequences (A, B, C and D) against the negative intronic splicing silencer (ISS-N1) of survival motor neuron 2 (*SMN2*), downstream of exon 7, that differed in the binding site compared to the already validated MO-10-34. The MO A and MO B sequences have perfectly complementary sequences to their target site, while only the first 10 nucleotides of MO C and MO D perfectly match with the target sequence within ISS-N1.

**Figure 2:**
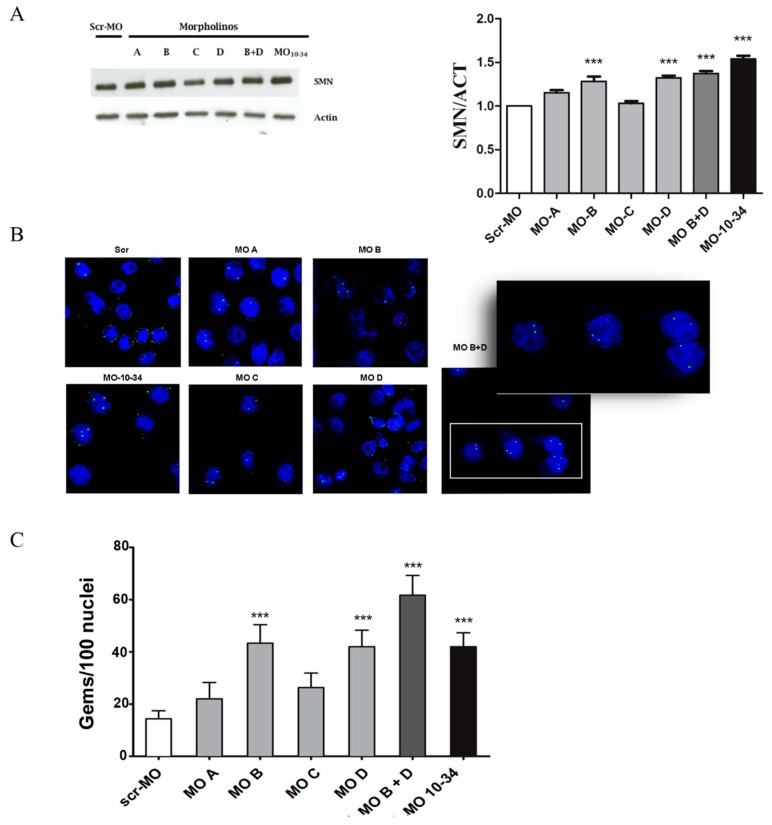
New MO sequences upregulate the SMN protein in HeLa cells. (**A**) A representative image of western blot analysis and densitometry analysis on HeLa cells treated with different MO sequences shows that the SMN protein levels increased after nucleofection with MO B and MO D. The combined treatment appeared to more efficiently increase SMN levels (*** *p* < 0.001) and was closer to the efficiency of MO-10-34 treatment compared to the other sequence alone (MO-10-34 vs. MO B + D *p* < 0.05; MO-10-34 vs. MO D *p* < 0.01; MO-10-34 vs. MO A, C and B *p* < 0.001); (**B**) Representative images of treated HeLa cells are shown. Nuclear gems (green) were detectable in all MO-treated cells compared to cells treated with scr-MO treated. In the right panel a magnification of the white frame of MO B + D image. Nuclei are labeled with diamidino-2-phenylindole dihydrochloride (DAPI) in blue; (**C**) Quantification results of the number of gems in HeLa cells treated with different MO sequences are shown. The greatest increase was observed after co-treatment with MO B + MO D (*** *p* < 0.001). Two independent observers examined at least 100 nuclei in randomly selected fields from each slide, and recoded the gems number. The values show the means + SD from 3 independent experiments. Scr-MO: scrambled-MO (white bar). Scale bar: 75 µm. MO B + D: 85 µm, insert 50 µm.

We have already demonstrated that the SMN protein levels can be upregulated by modifying *SMN2* splicing through targeting ISS-N1 with MO, particularly the MO-10-34 sequence [11]. To further improve the results obtained and the efficacy of our therapeutic approach, we designed four different MO oligomers targeting different sequences within the ISS-N1 region (Figure 1). First, we tested the efficiency of the four new MO sequences to increase SMN levels in human wild-type cells. HeLa cells were nucleofected with 20 µg of MO or scrambled (scr)-MO, as suggested by the manufacturer’s protocol, for western blot and immunocytochemistry analysis. We observed that all the MO sequences were able to increase the levels of the SMN protein in treated HeLa cells that were analyzed 72 h after treatment (Figure 2A). However, the MO B and MO D sequences were the most efficient compared to cells treated with scr-MO as a negative control (Figure 2A, *p* < 0.01). Therefore, we treated HeLa cells using MO B in combination with MO D (10 µg + 10 µg) and observed that the combined treatment appeared to efficiently increase SMN levels (Figure 2A, *p* < 0.01) and was closer to the efficiency of MO-10-34 treatment compared to the other sequence alone. 

The data obtained by western blot analysis were confirmed by immunocytochemistry analysis to detect the formation of nuclear complexes formed by SMN aggregates and other proteins, called nuclear gems, which are essential for pre-mRNAs splicing (Figure 2B). All the tested sequences increased the number of nuclear gems (Figure 2C). Interestingly, cells treated with MO B + MO D showed a greater increase in the formation of gems, even compared to what was observed in cells treated with MO-10-34 (*p* < 0.001, Figure 2C). 

### 2.2. Upregulation of SMN in SMA iPSCs

**Figure 3:**
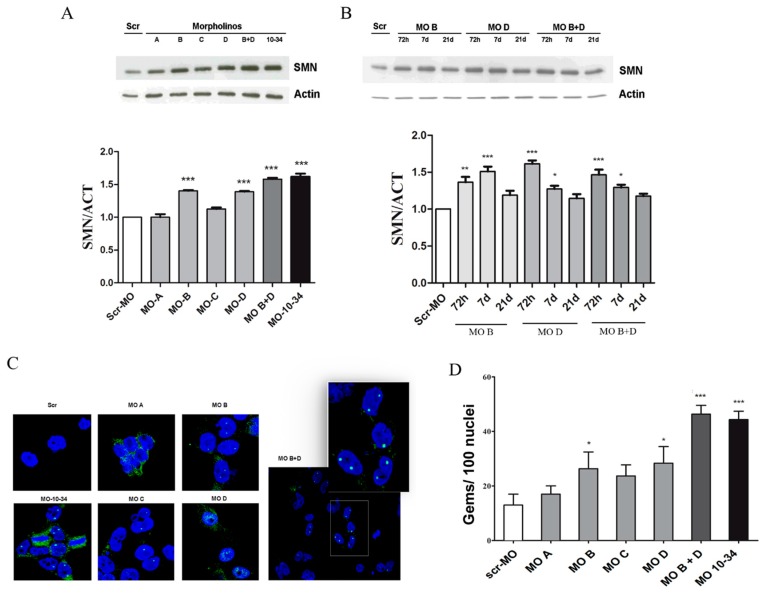
New MO sequences are able to upregulate the SMN protein in spinal muscular atrophy (SMA) induced pluripotent stem cells (iPSCs). (**A**) A representative image is shown of western blot analysis and densitometry analysis on iPSCs generated from a SMA type 1 (3 *SMN2* copies) patient treated with different MO sequences. The levels of the SMN protein increased significantly after treatment with MO B and MO D. Combined nucleofection with MO B + MO D was more effective and comparable to that of MO-10-34 treatment (*** *p* < 0.001); (**B**) A representative image is shown of western blot analysis and densitometry analysis on SMA iPSCs collected at different time points after the nucleofection (72 h; 7 days; 21 days, * *p* < 0.05, ** *p* < 0.01, *** *p* < 0.001); (**C**) Representative images are shown of treated SMA iPSCs. Nuclear gems (green) were detectable in all MO-treated cells compared to cells treated with scr-MO. In the right panel a magnification of the white frame of MO B + D image. Nuclei are labeled with DAPI (blue); (**D**) The number of gems are compared in human SMA iPSCs treated with different MO sequences. The greatest increases were observed after co-treatment with MO B + MO D or with MO-10-34 (*** *p* < 0.001). Two independent observers examined at least 100 nuclei in randomly selected fields from each slide, and recoded the gems number. The values show the means + SD from 3 independent experiments. Scr-MO: scrambled-MO. Scale bar: 75 µm. MO B + D: 85 µm, insert 50 µm.

As an in vitro model of the disease, we used human induced pluripotent stem cells (iPSCs) already generated in our laboratory from SMA patient fibroblasts through a non-integrating reprogramming protocol [12]. SMA iPSCs were nucleofected with the four MO sequences as described for HeLa cells. Western blot analysis confirmed that MO B and MO D were the most effective among the tested sequences and that the combined treatment with MO B + MO D was more effective than treatment with a single sequence (Figure 3A, *p* < 0.001). 

Cells treated with the most effective sequences (MO B, MO D or MO B + MO D) were collected at different time points after treatment (72 h, 7 days, 21 days) to assess if the splice-correction effect of MO oligomers was maintained after several replication cycles (Figure 3B). The levels of SMN in MO-treated iPSCs slightly decreased over time, although at 21 days after nucleofection, they were still upregulated compared with cells following scr-MO treatment (Figure 3B). No significant differences were observed after 72 h and 7 days in terms of stability between MO-10-34 and new MOs sequences (data not shown). Data obtained by western blot analysis were confirmed by detection of the number of nuclear gems by immunocytochemistry analysis (*p* < 0.001, Figure 3C,D). Furthermore, gem counts indicated a slight superiority of MO B + MO D over MO-10-34 (Figure 3D).

### 2.3. Effect of Mos on SMN Levels in the Central Nervous System of SMA Mice

**Figure 4:**
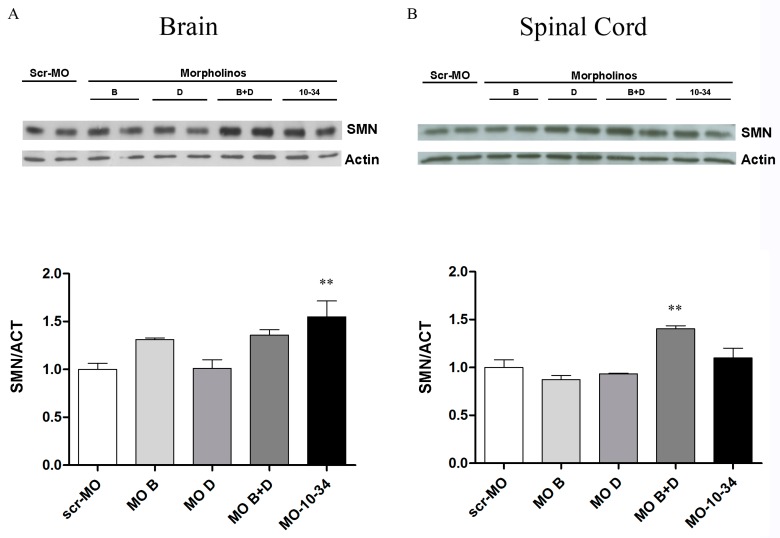
New MO sequences are able to upregulate the SMN protein in heterozygous SMA mice. (**A**) A representative image of western blot and densitometry analyses of brain samples from heterozygous SMA mice treated with different MO sequences shows that the efficacy of co-administration of MO B + MO D was high, even if not as high as MO-10-34 (** *p* < 0.01); (**B**) A representative image of western blot and densitometry analyses of spinal cord samples from heterozygous SMA mice treated with different MO sequences shows that co-administration of MO B + MO D was more efficacious than treatment with other sequences, even more so than treatment with MO-10-34 (*p* < 0.01). The values represent the means + SEM. Scr-MO: scrambled-MO.

To confirm the results obtained in vitro, we tested the most efficacious MO sequences in the SMA∆7 murine model. Heterozygous pups were treated with MO B, MO D, MO B + MO D, MO-10-34 or scr-MO by intracerebroventricular (ICV) injections at P1 and subcutaneous (SC) injections at P1 and P3 (12 nmoles total dose for each group), which is similar to the protocol used in our previous study [11]; brains (Figure 4A) and spinal cords (Figure 4B) were harvested at P7 and analyzed by western blot. In the brain, higher levels of SMN were detected by western blot analysis for all MO samples compared with scr-MO samples. Intriguingly, co-administration of MO B + MO D was the most effective treatment, even more than MO-10-34, in the spinal cord of treated mice (*p* < 0.01, Figure 4B).

## 3. Discussion

Numerous therapeutic approaches for SMA have been studied over the past few years. Correction of the SMN2 pre-mRNA splicing process with ASOs has been one of the most interesting and effective approaches, so much so that one of the ASOs, named Nusinersen, has been recently approved by the FDA/EMA (www.curesma.com). Nevertheless, this strategy needs to be further optimized and it is still unclear whether this approach can benefit all types of patients. 

MO is a variant of an ASO in which the phosphorothioate-ribose backbone, present in 2OMePS and in MOE oligomers, is replaced by a phosphorodiamidate-linked morpholine backbone, which makes it resistant to metabolic degradation. Because current ASO therapy requires repeated administration with an intrathecal injection, finding stable molecules with the same efficacy that do not require so many injections could be extremely important. In addition, MOs are virtually free of off-target effects because of their selective and short sequence specificity and their inability to interact with proteins. Interestingly, MO has been successfully tested in vivo both in SMA and Duchenne muscular dystrophy [10,13] where it has been shown to be potentially more effective and safe than the competing phosphorothioate ASOs in animal models [13]. Finally, we have already demonstrated that a 25-nt MO sequence targeting the ISS-N1 region of SMN2 (HSMN2Ex7D (-10-34)) led to robust neuromuscular and survival rescue in SMA mice, effectively increasing full-length SMN expression [11]. 

Because the splice-modulation efficiency of MOs is significantly influenced by its pairing target sequence and by the length of the MO sequence, selection of the best target sequence is a fundamental step for the optimization of MO efficacy. Furthermore, the effect of a combined administration of different MO sequences has never been explored.

We designed four new MO sequences to target the ISS-N1 region downstream of exon 7 in the *SMN2* gene, which were designed similar to our validated MO-10-34 sequence. The MO A and MO B sequence have a perfectly complementary sequence to their target site, while only the first 10 nucleotides of MO C and MO D perfectly match with the target sequence within ISS-N1. The unmatched tail of the two sequences could hamper exon 7 splicing by a steric block effect. Indeed, steric block caused by our MOs should prevent the splicing machinery from being assembled, thereby leading to exon 7 retention.

First, we tested the efficacy of the new sequences in vitro in wild-type human cells and in iPSCs obtained from SMA patients. As expected, the different MO sequences showed variable efficacy in correcting the splicing of the *SMN2* transcript and consequently increased the SMN protein levels. Among the new sequences, only MO B and MO D significantly increased SMN protein production. We also studied whether combined treatment with the two most promising MO sequences could show an additive effect and enhance the splice-correction efficacy of MO while maintaining the same total dosage. Combined treatment with MO B plus MO D was more efficient than treatment with a single MO sequence and, most importantly, was the only treatment able to significantly increase the number of gems, which are discrete bodies within which full length SMN is highly enriched and that are essential for pre-mRNA splicing. The combined treatment was also the only one comparable or even better than MO-10-34. Analysis of the SMN protein levels in MO-treated iPSCs collected at different time points after treatment showed that the correction effect was maintained, although a trend towards decreasing levels was observed. We tested the MO sequences that performed best in vitro and also tested combined MO B + MO D treatment in heterozygous SMA pups in comparison to MO-10-34 treatment. The results obtained in vivo confirmed that the combined treatment with MO B + MO D had a greater efficiency than treatments with MO B or MO D alone in increasing the SMN protein levels in the brain and in the spinal cord of heterozygous SMA mice. Interestingly, the efficacy of the combined treatment was greater than that of MO-10-34 in the spinal cord, which is the most affected tissue in SMA. 

Considering the design of MO B and MO D sequences, we speculate that the difference between the two sequences could be the reason for the enhanced efficacy observed. Indeed, the unpaired nucleotide tail of MO D could reasonably be responsible for an enhanced steric block effect of MO D itself and MO B, which perfectly matches the target sequence. 

Our results confirm that targeting the ISS-N1 region leads to efficient modulation of the *SMN2* transcript and indicate that optimization of the target sequence, even with small variations in the MO sequence, can achieve more efficient production of functional SMN protein, consequently ameliorating the SMA disease phenotype. Moreover, we observed that treatment with the combination of MO B + MO D increased the number of gems and SMN protein in the mouse spinal cord compared to treatment with MO-10-34. These data need to be further confirmed to assess the efficacy on survival and neuromuscular function in affected mice, but suggest that the use of combinations of different sequences of MOs can enhance their efficacy. 

## 4. Materials and Methods

### 4.1. Morpholino Oligomers

We designed four MO sequences (A, B, C, D) targeting the *SMN2* ISS-N1 region, downstream of exon 7 (Figure 1). The MO A and MO B sequence have a perfectly complementary sequence to their target site, while only the first 10 nucleotides of MO C and MO D perfectly match with the target sequence within ISS-N1. All the MO oligomers were synthesized by Gene Tools as Bare MO, without any further modifications (Available online: www.gene-tools.com). The scrambled (scr)-MO sequence was designed based on the best control sequence predicted by a bioinformatics tool (Gene Tools, available online: www.gene-tools.com). The powdered compounds were dissolved in sterile water at the appropriate concentration for in vivo and in vitro experiments as previously described [11].

The sequences used are the following:

MO-10-34: GTAAGATTCACTTTCATAATGCTGG
Scr-MO: GTAACATTGACTTTGATATTCCTGG
MO A: TCATAATGCTGGCAGAC
MO B: GTAAGATTCACTTTCATAATGC
MO C: GATTCACTGTCAGAAGGCTGGCAGAC
MO D: GATTCACTCTCACAACGCTGGCAGAC

### 4.2. Cell Cultures

HeLa cells were plated with Dulbecco’s modified Eagle’s medium (MEM)/F12 (Life Technologies; Carlsbad, CA, USA) supplemented with 15% fetal bovine serum (Life Technologies), 1% pen/strep (Life Technologies), 1% amphotericin B (Life Technologies). SMA type 1 iPSC lines were already available in our laboratory [14]. The iPSCs were maintained in culture on Matrigel-coated dishes with Essential 8 medium (Life Technologies).

### 4.3. MO Transfection

SMA type 1 iPSCs, three copies of *SMN2*, and HeLa cells were nucleofected with the Neon Nucleofection System (Life Technologies) following the manufacturer’s instructions. MO oligomers were added at a dosage of 20 μg for 5 × 10^6^ cells; for combined treatment, the same 20 μg dose was used (10 μg MO B + 10 μg MO D). Treated HeLa cells were harvested 72 h after treatment for immunohistochemistry and western blot analysis. Treated SMA iPSCs were harvested at different time points: 72 h, 7 days and 21 days after treatment. The results were derived from three independent experiments per condition.

### 4.4. Immunocytochemistry of HeLa Cells and iPSCs

Forty-eight hours after treatment, cells were fixed in 4% paraformaldehyde for 10 min, blocked with 10% BSA and permeabilized with 0.3% Triton X-100 in PBS. The primary antibody SMN (1:100, BD; San Jose, CA, USA) was maintained in 3% BSA solution overnight at 4 °C. The secondary antibody anti-mouse FITC-conjugated (1:100, Agilent; Santa Clara, CA, USA) was used. Cells were analyzed using a confocal LEICA LCS2 microscope. The number of gems was quantified as described previously [12]. Two independent observers examined at least 100 nuclei in randomly selected fields from each slide, and recoded the gems number. The results were derived from three independent experiments per condition.

### 4.5. Western Blot

Western blot analysis was performed as previously described [15]. Briefly, cells were sonicated on ice for 10 min in lysis buffer supplemented with protease and phosphatase inhibitors (Pierce Rockford, IL, USA). 20 mg of frozen tissue was homogenized in 0.4 mL (brain) or 0.2 mL (spinal cord) of 1 × protein sample buffer containing 2% (*w*/*v*) SDS, 10% (*v*/*v*) glycerol, 50 mM Tris-HCl (pH 6.8), and 0.1 M DTT. Five micrograms of protein were separated by 12% SDS-PAGE and electrophoretically transferred to a nitrocellulose membrane. Membranes were incubated with anti-SMN (1:1000, BD) and anti-actin (1:1000, Sigma, St. Louis, MO, USA) antibodies. After labeling with peroxidase-conjugated secondary antibody (Life Technologies), the proteins were detected using a chemiluminescence assay (Amersham, Pittsburgh, PA, USA). Densitometry analysis was performed using Image J software (Softonic, Barcelona, Spain, www.imagej.en.softonic.com).

### 4.6. Animal Procedures

All transgenic animals were purchased from the Jackson Laboratory (Bar Harbor, ME, USA). All animal experiments were approved by the University of Milan and Italian Ministry of Health review boards (1007/2016-PR, 21/10/2016), in compliance with US National Institutes of Health Guidelines [11].

The SMAΔ7 transgenic model was used [16]. Heterozygous unaffected mice (Smn^+/−^, hSMN2^+/+^, SMNΔ7^+/+^) were bred and pups were genotyped as previously reported [11]. Heterozygous pups (*n* = 4 per group) were cryoanesthetized and injected as previously described [11]: 12 nmoles per injection of each MO were administered into the cerebral lateral ventricle at postnatal day 1 (P1) and subcutaneously at P1 and P3. For the combined administration, 6 nmoles MO B + 6 nmoles Mo D were injected. Treated animals were sacrificed at P7 and the brain and spinal cord were harvested for western blot analysis.

### 4.7. Statistical Analysis

All statistical analyses were performed using the Prism software. The gems/nuclei ratios that were obtained by immunocytochemistry analysis and the data from western blot analysis were analyzed using the one-way ANOVA test with post-hoc Tukey’s test for multiple comparison. A *p* value < 0.5 was considered statistically significant.

## Abbreviations

SMA	Spinal Muscular Atrophy
SMN	Survival Motor Neuron
ASO	Antisense oligonucleotide
MO	Morpholino
IPSCs	Induced pluripotent stem cells
MN	Motor neuron
SCR	Scrambled
ICV	Intracerebroventricular
SC	Subcutaneous
